# The Western Ophthalmologic and Oto-Laryngologic Association

**Published:** 1899-03

**Authors:** 


### The Western Ophthalmologic and Oto-Laryngologic Association.

The annual meeting of the Western Ophthalmologic and Oto-Laryngologic Association was held in New Orleans, February 10th and 11th. Owing to the unavoidable absence of the President, Dr. J. Elliott Colburn, of Chicago, the First Vice-President, Dr. W. Scheppergrell, of New Orleans, presided. Two joint sessions and three sessions of the Ophthalmologic and Oto-Laryngologic Sections respectively were held, and many important papers read and discussed.

The following officers were elected for the ensuing year: Dr. W. Scheppergrell, of New Orleans, president; Dr. M. A. Goldstein, of St. Louis, first vice-president; Dr. H. V. Wurdemann, of Milwaukee, second vice-president; Dr. E. C. Ellett, of Memphis, Tennessee, third vice-president; Dr. F. C. Ewing, of St. Louis, secretary; Dr. W. L. Dayton, of Lincoln, Nebraska, treasurer.

St Louis was selected for the next annual meeting.

The following names were added to the list of honorary members: Dr. Geo. Stevens, of New York; Dr. St. Clair Thompson, of London; Dr. R. Coen, of Vienna, Austria; Dr. E. J. Moure, of Bordeaux, France; Dr. J. Sendziak, of Warsaw, Russia; Dr. Marcol Natior, of Paris, France; Dr. C. Ziem, of Dantzig, Germany; Dr. A. A. Cuye, of Amsterdam, Holland.

The new members elected were as follows: Dr. J. A. Caldwell, of McKinney, Texas; Dr. O. Joachim, of New Orleans; Dr. W. H. Baldinger, of Galveston, Texas; Dr. J. S. Nott, of Kansas City, Mo.; Dr. J. S. Lichtenberg, of Kansas City, Mo.; Dr. J. W. Bettengen, of St. Paul, Minn.; Dr. J. W. Chamberlin, of St. Paul, Minn; Dr. H. M. Starcky, of Chicago; Dr. R. Brunson, of Hot Springs, Ark.; Dr. Max Thorner, of Cincinnati; Dr. J. W. Scales, of Pine Bluff, Ark.; Dr. E. M. Singleton, of Marshalltown, Ia.; Dr. F. C. Ewing, of St. Louis.

